# Explorations to improve the completeness of exome sequencing

**DOI:** 10.1186/s12920-016-0216-3

**Published:** 2016-08-27

**Authors:** Chen Du, Barbara N. Pusey, Christopher J. Adams, C. Christopher Lau, William P. Bone, William A. Gahl, Thomas C. Markello, David R. Adams

**Affiliations:** NIH Undiagnosed Diseases Program, Common Fund, National Institutes of Health, National Human Genome Research Institute, Bethesda, MD USA

**Keywords:** Clinical exome sequencing, Analytical quality, Performance enhancement, Clinical genomics, Rare diseases, Completeness problem, False negative results

## Abstract

**Background:**

Exome sequencing has advanced to clinical practice and proven useful for obtaining molecular diagnoses in rare diseases. In approximately 75 % of cases, however, a clinical exome study does not produce a definitive molecular diagnosis. These residual cases comprise a new diagnostic challenge for the genetics community. The Undiagnosed Diseases Program of the National Institutes of Health routinely utilizes exome sequencing for refractory clinical cases. Our preliminary data suggest that disease-causing variants may be missed by current standard-of-care clinical exome analysis. Such false negatives reflect limitations in experimental design, technical performance, and data analysis.

**Results:**

We present examples from our datasets to quantify the analytical performance associated with current practices, and explore strategies to improve the completeness of data analysis. In particular, we focus on patient ascertainment, exome capture, inclusion of intronic variants, and evaluation of medium-sized structural variants.

**Conclusions:**

The strategies we present may recover previously-missed, disease causing variants in second-pass exome analysis. Understanding the limitations of the current clinical exome search space provides a rational basis to improve methods for disease variant detection using genome-scale sequencing techniques.

**Electronic supplementary material:**

The online version of this article (doi:10.1186/s12920-016-0216-3) contains supplementary material, which is available to authorized users.

## Background

The Undiagnosed Diseases Program (UDP) of the National Institutes of Health (NIH) was established in 2008 to evaluate patients who were undiagnosed despite an extensive medical workup [1–3]. Besides thorough clinical phenotyping by multiple specialists, the UDP has been utilizing exome sequencing and SNP array analysis when a genetic etiology is suspected. The feasibility of using exome sequencing to identify new disease genes was first demonstrated in 2009 [4, 5] and has ever since contributed to the discovery of many Mendelian disease genes [6]. Exome sequencing studies have become an increasingly routine clinical approach with a reported 25 % molecular diagnostic rate [7, 8]. In the past few years, we observed that an increasing percentage of pediatric patients referred to the UDP has already been studied with a clinical exome. Hence, we employed an extended set of analytic approaches to identify disease-causing variants beyond those detected by current clinical exome analysis.

Several specific features of an exome analysis pipeline contribute to the final sensitivity and specificity of the overall test. They include the design of the analysis, e.g., included family members, limitations of the underlying sequencing technology, and a number of specific analytic parameters that affect variant filtration and prioritization. These include segregation rules, application of allele frequency cutoffs derived from control populations, and predictions of deleteriousness [9, 10]. The choice of analytic parameters depends on the experimental design and scope of testing, with an overall goal of optimizing the final list of prioritized variants to be subjected to manual curation. Stringent parameters, focusing only on coding sequences in known disease genes and common Mendelian inheritance models, may filter out a “true” variant and create false negative results. This approach is commonly applied in clinical exome analysis where clinical interpretability is prioritized over new gene discovery. Conversely, relaxed filtration settings feature enhanced sensitivity but generate a number of false positive variants that increase the work associated with final curation. This approach is generally more suitable to a research-level analysis, where variants in genes not yet associated with disease may be of intrinsic interest.

Our current goal is to optimize research-level exome analysis for single, small-pedigree families. We quantify the consequences of widening the final search space using several analytic techniques. They include sequencing additional family members; evaluation of minimal exome coverage; reducing the number of false negative results by considering variants in non-coding regions; and searching for medium-sized indels missed by the standard genotyping modules of current analytical pipelines. Each of these potential second-pass procedures can be employed when standard exome analyses fail to provide a satisfactory explanation for the patient’s clinical features.

## Results

### Patients

**Fig. 1:**
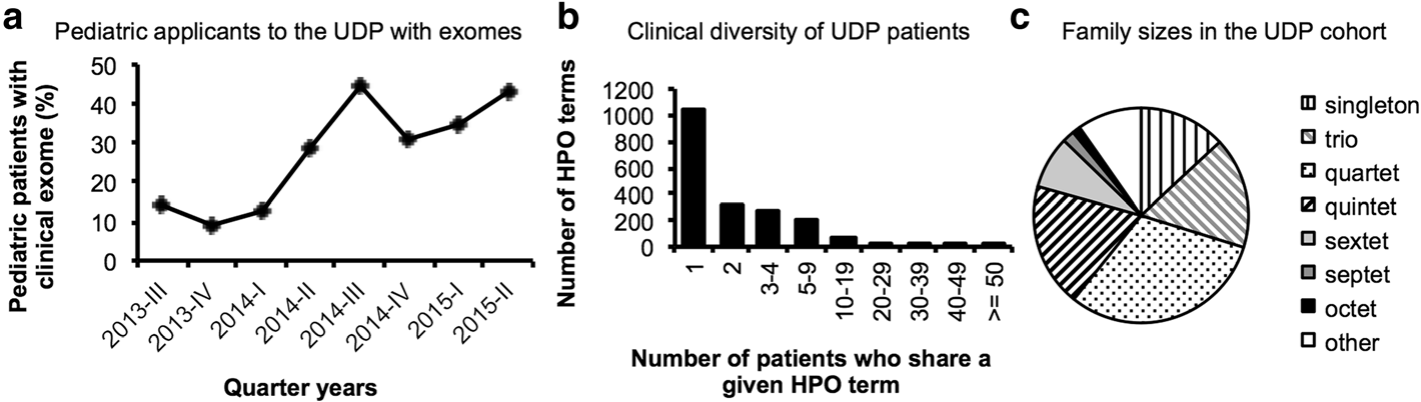
Characteristics of the UDP patient cohort. **a** Percentage of pediatric cases (n = 11-28 per quarter year) with prior inconclusive exome sequencing that applied to the UDP. **b** Number of HPO terms that are shared by a given number of patients (total *n* = 350 affected individuals). The top five HPO terms that were used in more than 50 patients were spasticity, global developmental delay, gastroesophageal reflux, seizures and short stature. **c** Family structures of nuclear families in the UDP cohort, *n* = 329

A growing number of pediatric patients referred to the UDP present with prior unrevealing clinical exome studies (Fig. 1a), which necessitates the development of novel diagnostic strategies to improve experimental power. Cohort-based studies, which have been successful in detecting disease genes by exome sequencing [4, 5], are not an appropriate tool to employ for our subjects given their high degree of phenotypic diversity as demonstrated by comparison of Human Phenotype Ontology terms [11, 12] that were used to characterize each affected patient (Fig. 1b). Additionally, most of our cases are limited to a nuclear family that is too small to achieve the LOD score thresholds used in linkage analyses (Fig. 1c). These factors have focused our attention on the general problem of maximizing the information that can be extracted from small-family, *n = 1* cases [13].

### Ascertainment of family members

Our study participants have undergone extensive medical testing before being accepted into our program. As a result, many easily-identifiable diagnoses, and interpretable DNA sequence variants, have been previously excluded in patients of the UDP cohort, in particular when patients had undergone prior clinical exome testing. Therefore, our patients require an exome sequencing strategy optimized for *agnostic* testing, going beyond a clinical routine analysis pipeline, which is optimized for interpretable results and identifying variants in known disease genes.

A typical exome analysis pipeline includes an unsupervised variant filtration component. This may be followed by manual BAM file inspection, manual bioinformatic curation and expert clinical evaluation. Practice varies with regard to the number of family members included in exome sequencing and analysis. The value of family trios is increasingly recognized [8, 11]; sequencing of all available siblings has been a common practice in the UDP [14].

**Fig. 2:**
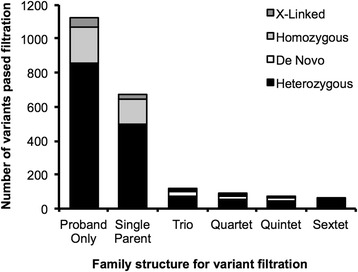
Effects of family members on the number of variants returned by computerized filtration. Variants of 36 quintets and 9 sextet families were analyzed with an increasing number of family members. Bars show average number of variants that passed a basic filtration algorithm for segregation with disease, population frequency and transcript effects, based upon various inheritance models

To study the effect of included family members on exome analysis, we performed a standard variant filtration analysis on 45 families, while varying the family composition. These families included the proband, both unaffected parents and at least two additional siblings (at least a quintet in total). Taking different numbers of family members into account (Fig. 2), we filtered variants for segregation with disease, population frequency and transcript effects, but *did not* filter for known disease genes, *in silico* predictions of deleteriousness, or family-based linkage. The difference in the number of variants returned for manual evaluation was most striking between a singleton and a trio analysis, with about 1,126 (range 886-1521) vs. 117 (range 59-265) variants passing automated filtration, similar to the 10-fold reduction reported previously [11]. A large part of this effect is attributable to a decrease in the number of heterozygous variants. In a trio, such variants could be assessed for *de novo* occurrence in light of parental genotypes and/or phased correctly as compound heterozygous variant pairs. The number of false positive homozygous recessive variants was greatly reduced by the awareness of parental genotypes. Additional number of siblings beyond a trio provided a further reduction in the average number of variants down to 88 (range 22-209), 69 (range 8-171) and 54 (range 11-109) for quartets, quintets and sextets, respectively. These reductions were obtained even without mapping recombination sites [15], by which siblings can exclude genomic regions from consideration and therefore remove large numbers of false positive variants.

In our research setting, the increased filtration power of additional informative meioses in family members balanced against the effort required to obtain the correct affected status, and the financial cost of sequencing and data processing. We justified this approach as being worthwhile in order to reduce the number of *false positive* variants when analyzing individual families (*n = 1*), so that time and effort could instead be used to reduce the extent of *false negative* results and therefore to improve completeness of analysis.

### Exome coverage

Capture and enrichment of the exome for medical sequencing limits most of the analysis to the protein coding sequences –about 1-2 % of all 3.2 billion base positions in the human genome– where the majority of previously documented disease-causing variants occur [16]. To assess loss of data due to lack in exome capture, we selected a subset of exomes that included 54 probands of the UDP cohort. Each member of this subset was sequenced under the same conditions at one sequencing center, and aligned through the same pipeline.

**Fig. 3:**
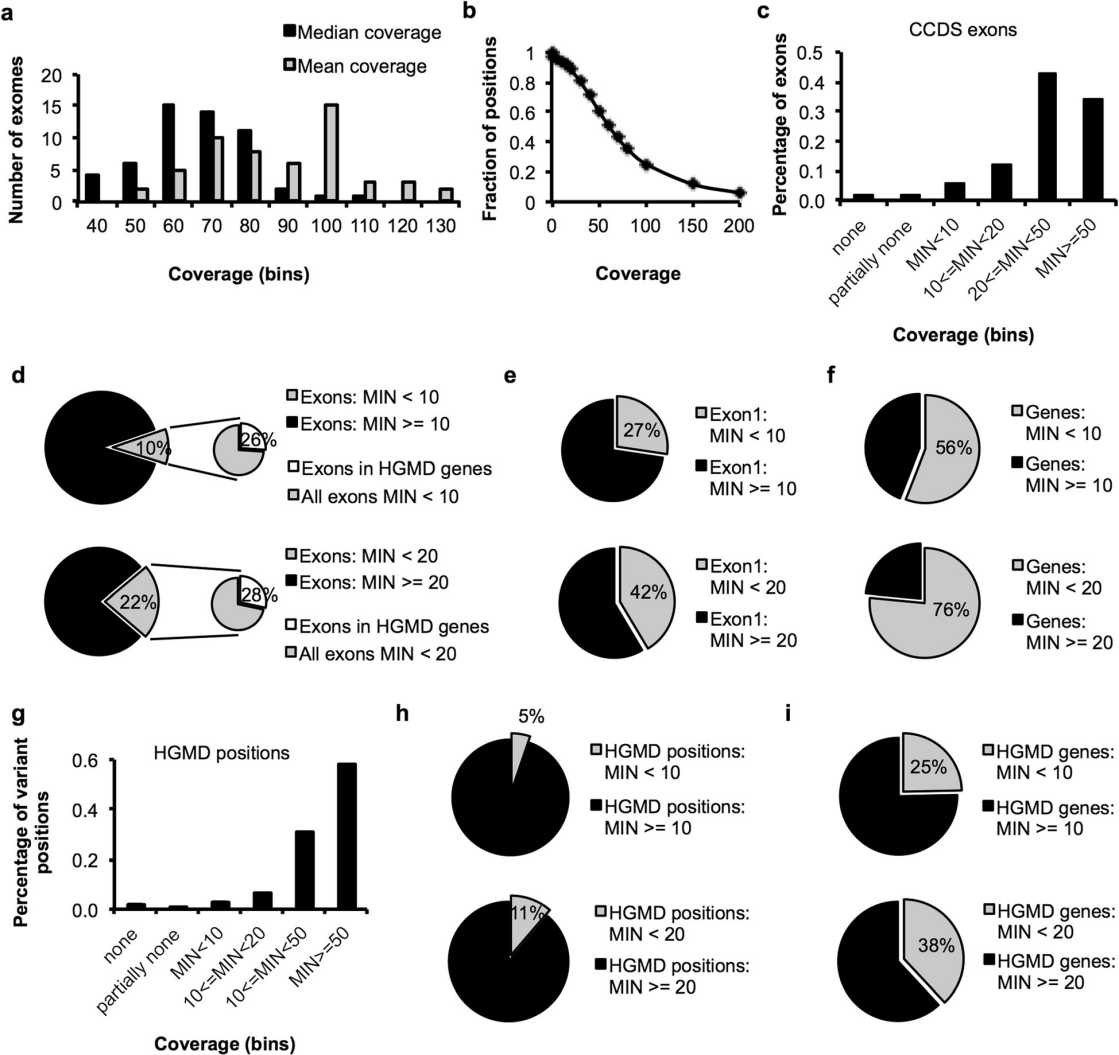
Potential false negative space due to lack of coverage. **a** Mean and median coverage based on exonic CCDS positions (total about 31.9 Mb) in 54 exomes of probands used for coverage analysis. **b** Fraction of exonic positions as a function of coverage. **c-f** Coverage was based on the minimum read depth that occurred in CCDS exons, grouped by exons (total about 190,000), first exons (total about 20,600) or genes (total about 18,400). **g-i** The minimum number of mapped reads at each position of known HGMD variants (classes “DM” and “DM?”) was determined by SAMtools and grouped by variant positions (total about 118,900) or genes (total about 4,000)

When coverage is based on the targeted regions as determined by the capture kit (TruSeq, about 61 Mb), we observed a mean coverage of 76 reads and a median coverage of 57 reads. 85 % of targeted positions were covered more than 20×, a coverage frequently used as a minimal read depth requirement for confident genotyping of both alleles. Since capture kits differ in their target regions, we evaluated the coverage of all exonic regions defined by CCDS (about 31 Mb), as a measure of desired capture. Here we observed a mean coverage of 82 reads (range 44-130) and a median coverage of 61 reads (range 32-110), with an average of 88 % (range 74-94) of exonic positions covered more than 20× (Fig. 3a, b).

Next we examined coverage based on CCDS *exons* instead of individual *positions* and grouped all exons into categories based on the minimum number of reads occurring in a given exon. This analysis showed that an average of 2.2 % of exons (range 1.6-2.8) had no coverage at all, 2.1 % (range 1.6-2.8) of exons had partially no coverage, 6 % (range 2-17) showed a minimum coverage of fewer than 10 reads, and 12 % (range 2-32) fewer than 20 reads (Fig. 3c). In total, an average of 10 % of exons (range 6-21) had a minimum coverage of fewer than 10 reads and 22 % (range 8-53) of exons had a read depth of fewer than 20 reads in at least one position (Fig. 3d). Of these low coverage exons, an average of more than 25 % (range 24-30) were in genes known to harbor disease-causing or likely disease-causing variants (HGMD classes “DM” and “DM?”). In addition, coverage below 10 reads and below 20 reads was observed in 27 % (range 19-38) and 42 % (range 26-63) of first exons, respectively (Fig. 3e), indicating a notable contribution of first exons to the low coverage regions of exon sequencing, as generally recognized. When analyzed by entire genes (total number *n* = 18.351 for females, *n* = 18.409 for males), an average of 56 % (range 39-78) and 76 % (range 51-94) of genes had a minimum coverage below a depth of 10 and 20 reads, respectively (Fig. 3f), suggesting that low coverage occurred across the entire length of genes.

To estimate the impact of insufficient coverage on the diagnosis of known diseases, we queried the read depth at positions of known disease-causing variants listed in HGMD (*n* = 118.861 positions for females, *n* = 118.949 positions for males). This subset of positions showed a higher coverage with only 5 % (range 3-11) of known disease-causing variant positions covered less than 10×, and 11 % (range 4-31) were covered with fewer than 20 reads (Fig. 3g-i). Low coverage variant positions of a read depth of less than 10× occurred in about 25 % (range 17-38) of all HGMD genes and in 38 % (range 22-62) based on a coverage of less than 20 × .

In a next step we analyzed the consistency of coverage across proband samples. Of 64,818 autosomal exons that showed read depths below 10 reads, 8199 exons were affected by low coverage in all 54 probands, of which 4406 exons were within the target sequence of the capture kit used (list of exons in Additional file 1: Table S1). When analyzed by genes, 1304 genes were covered with fewer than 10 reads in all 54 probands in more than 25 % of their exons. In these genes, all low coverage exons in 841 genes were within the targeted region of the capture kit used (list of genes in Additional file 1: Table S2). Coverage below 10 reads in *all exons* occurred in 409 genes, of which 277 genes consisted of a single exon only.

While it is possible to characterize the number of variants in known disease genes that are missed due to incomplete capture, we hypothesize that variants in genes not yet associated with disease may be missed due to lack of coverage of one of the two alleles, especially if strict requirements for read depth are applied during data analysis.

### Non-exonic variants

The majority of reported disease-causing variants reside in coding regions or canonical splice sites at exon/intron boundaries [16] but there are instances where deep intronic variants have been associated with disease [17–19] and genome sequencing has revealed evidence of selective pressure on intergenic and intronic regions, suggesting functional conservation [20]. Targeted re-analysis of candidate loci for non-coding variants has been successfully attempted [21]. However, non-coding variants are also detected as a result of off-target capture, and may be of high quality [22]. Therefore we quantified the analytical potential of targeted and off-target non-exonic variants, while exploring strategies to optimize the less-favorable signal to noise characteristics.

**Fig. 4:**
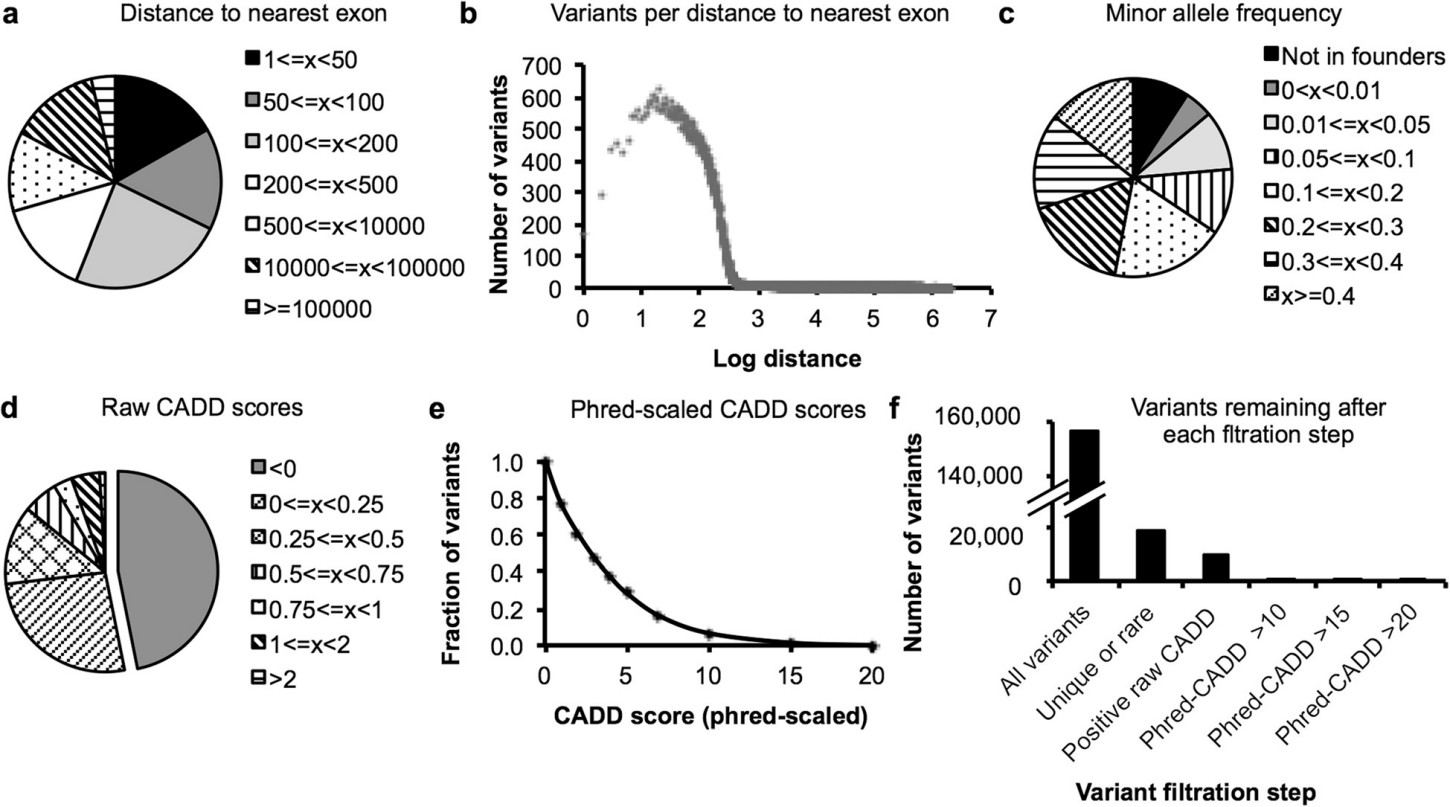
Prioritization of non-exonic variants. Non-exonic variants of 54 probands and their parents were evaluated. **a** The distance of non-exonic variants to the nearest exon was determined based on UCSC exon regions and the average number of variants was grouped by distance. **b** The average number of variants observed at a given distance to the nearest exon plotted over distance on a logarithmic scale. **c** Minor allele frequencies estimated by a founder population comprised of 106 parents, excluding each proband’s own parents. Number of variants was averaged and graphed per allele frequency group. **d** CADD scores [23] were calculated and the average number of non-coding variants were grouped by raw CADD score. **e** Fraction of variants as a function of Phred-scaled CADD scores. **f** Average number of non-coding variants remaining for consideration after each filtration or prioritization step

We analyzed variants called in 54 probands and their parents in non-exonic locations, defined as outside the UCSC exon regions (Fig. 4). On average, about 156,000 non-exonic variants were called in each proband (range from about 121,000 to 252,000). The median distance to the nearest exon was 169 bases (range 142-298) and the mean was 15,000 bases (range 11,600-25,700), with a maximal distance observed of 1.7 million bases on average. About 70 % of variants were within a distance of 500 bases from the nearest exon boundary (Fig. 4a). The number of non-exonic variants dropped consistently at a distance of about 300-500 bases from the nearest exon (2.47 to 2.69 on the logarithmic scale in Fig. 4b). Given an average DNA libraries size of 280 bp, this cutoff appears to correspond to the maximal distance between a non-coding variant and targeted exonic sequence that occurred on either end of the same DNA fragment. This suggests that most non-exonic variants were sequenced as flanking regions of intended capture, rather than captured due to sequence similarity or presented as artifacts due to misalignment.

To filter the large number of non-coding variants, we estimated the minor allele frequencies based on a founder population comprised of 106 parents. We found that only 6 % of the variants seen in each proband were absent from the founder population, which excluded each proband’s own parents (Fig. 4c). Of variants present in the founder population, only 6 % were rare at a 1 % lower confidence interval limit of the estimated minor allele frequency. Overall, variant filtration based on allele frequency reduced the number of unique and rare non-exonic variants to 19,000 per proband on average (range from 7,500 – 89,000).

Prediction of deleteriousness is particularly difficult for non-coding variants since most prediction tools are limited to non-synonymous codon changes or canonical splice site positions. We tested the use of CADD scores [23] on our non-coding variant set, since this approach allows scoring of *all* SNVs and CNVs, not limited to coding variants. Raw CADD scores below zero were returned for 47 % of all non-coding variants, indicating that these variants were not different from known benign variation (Fig. 4d). When we analyzed Phred-scaled CADD scores, we observed a mean score of 3.82, median of 2.84, a minimum score of 0.001 and a maximum of 36.5 on average. Only 7 % of variants obtained a score higher than 10 and only 1 % higher than 15 (Fig. 4e), indicating that a very low number of non-coding variants were actually predicted deleterious by CADD scores. Therefore, CADD scores could be an approach to highlight potentially interesting variants within the vast pool of mostly benign, but poorly annotated non-coding variants. More focused tools to predict splice site changes using multiple different algorithms and that can be incorporated into an automated computational pipeline should be able to prioritize additional variants for consideration.

Taking all information together, filtration based on allele frequency reduced the number of variants from initially 156,000 non-exonic variants per proband to 19,000 (Fig. 4f). Using a conservative approach to predict benign variants by negative raw CADD scores reduced the number of remaining variants to 9,400. Applying prediction of deleteriousness, an average of 1,200 variants were predicted damaging at a Phred-scaled CADD score of 10. When more stringent filters were applied, only 285 variants on average obtained a CADD score higher than 15 and only 40 variants on average scored higher than 20. Depending on the desired stringency, we found that these filtration strategies generated a tractable number of additions to a routine second pass analysis.

### Medium-sized indel calling

The limitations of genome-scale data analysis to identify structural variants is another known cause of false negative results. Standard variant callers typically identify indels up to about 50 bases. Supplementing exome diagnostics with SNP chip or array-CGH data is known to detect indels larger than a few kilobases genome wide. Therefore, a large range of medium-sized indels from 50 bases to a few kilobases remain unaccounted for in subsequent variant evaluation pipelines, contributing to incomplete analyses.

Many attempts have been made to address this issue by calling indels from exome sequencing data with additional methods [24–28], and such efforts have recently been implemented in large scale exome research studies [11]. While some programs examine read depth against a reference population, Pindel [29] extracts unmapped reads from BAM files and analyzes soft clipped bases of read pairs for evidence of medium-sized structural variation. We used Pindel to quantify the extent of incomplete analysis resulting from missed indels in a cohort of 54 probands and their parents.

**Fig. 5:**
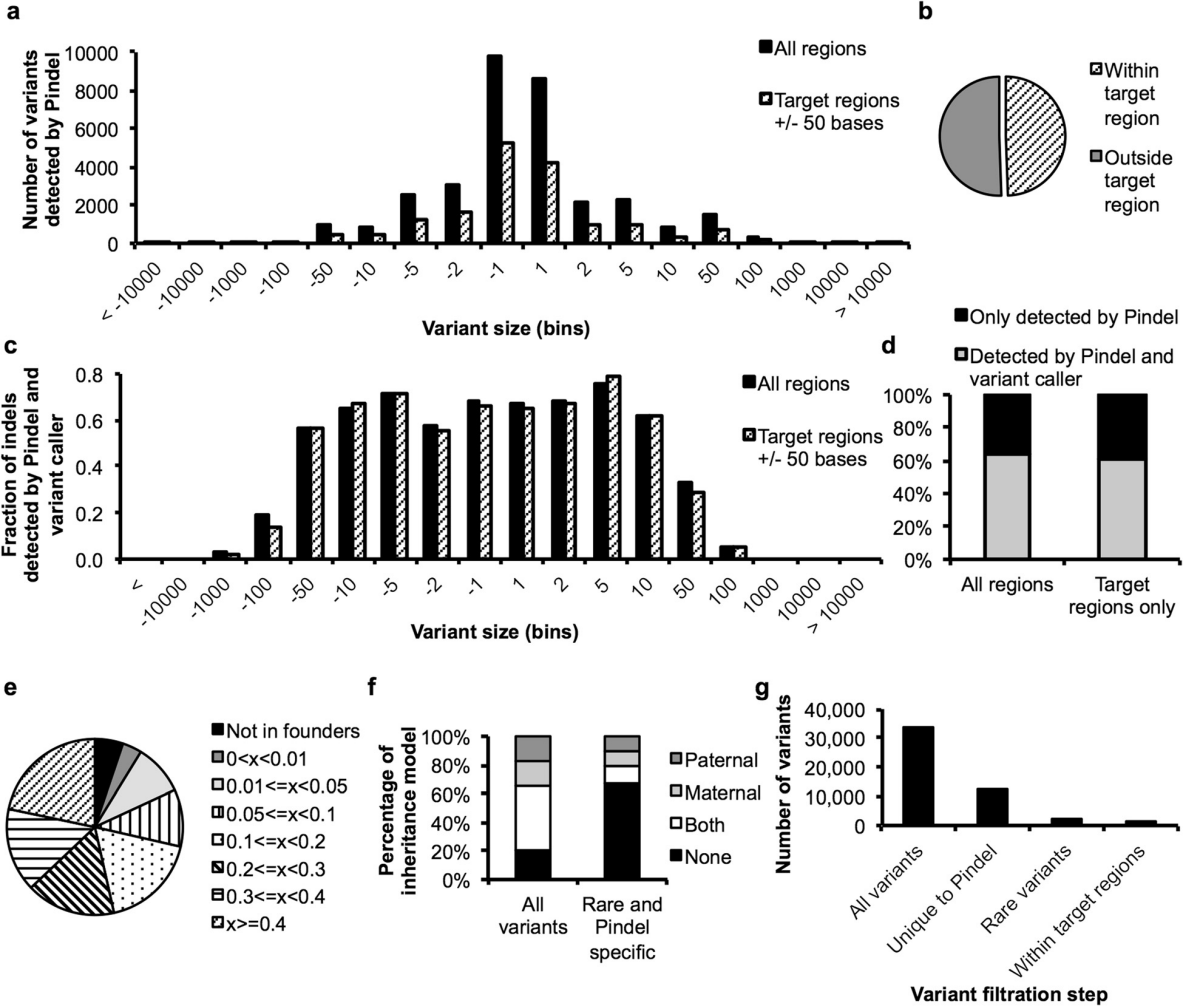
Calling medium sized indels with Pindel. Pindel [29] was used to detect structural variants in exome sequencing data of *n* = 54 probands. **a** Average number of variants by size detected by Pindel within the target region +/- 50 bases or in all regions. **b** Percentage of variants within or outside the target region. **c** Comparison of Pindel variants to the number of indels called by the variant caller broken down by variant size. **d** Percentage of Pindel variants within or outside the target regions +/- 50 bases that were also called by the variant caller. **e** Estimated allele frequencies based on a founder population of 106 parents. **f** Phasing of variants detected in the probands considering all variants or only variants that were unique to Pindel and that were rare. **g** Average number of Pindel variants after each step of filtration left for continued evaluation

Pindel detected on the order of 33,000 structural variants per proband on average (range about 22,000 to 54,000), of up to +/- 16,000 bases in size (Fig. 5a). Half of these indels occurred within the target region defined by the capture kit and 50 bases of flanking regions (Fig. 5b). On average, 63 % of all indels and 61 % of indels within target regions were also detected by the variant caller and were therefore redundant (Fig. 5d). A breakdown by size showed that deletions of fewer than 50 bases and insertions of fewer than 10 bases were identified by both Pindel and the genotype caller in 56 % to 76 % of variants (Fig. 5c). The overlap of called variants dropped rapidly beyond deletions larger than 100 bases and insertions larger than 50 bases.

We also used Pindel to call indels in the probands’ parents and used their variants to estimate allele frequencies. This analysis revealed that only 5 % of indels in the probands were absent from the control population (Fig. 5e). Of the indels that were present in the founder population (excluding each proband’s own parents), 8 % were rare at a 1 % lower confidence interval limit of the estimated minor allele frequency. Thus, filtering all Pindel variants by allele frequency for unique or rare variants reduced the number of variants to about 4,200 on average (range from 1,500 – 11,700).

When we analyzed phasing of all Pindel variants of the proband, about 34 % of all variants appeared to be inherited by one of the parents, 46 % occurred in both parents and 20 % could not be associated with an inheritance pattern (Fig. 5f). As expected, in a subset of variants that are only detected by Pindel and that are rare in or absent from the founder population, the percentage of variants that are present in both parents is greatly reduced (12 %), and enriched for variants not detected in the founder population (68 %).

Combining all information gained on the indels called by Pindel (Fig. 5g), we started with an average of 33,000 Pindel variants per proband. An average of 12,200 variants were detected by Pindel only and not by the standard variant caller, of which an average of 2,600 variants were rare or unique indels in the proband by filtration based on founder frequencies (of which 1,200 variants map within target regions). This appears to be the lower limit that purely frequency-based bioinformatics tools can reach based on this cohort size, before variants are evaluated for other parameters such as Mendelian inheritance models and prediction of deleteriousness. With increased numbers and matched ethnicity of individuals in the control population, the power of filtration of ancient and benign variants based on allele frequency will improve and optimize computerized prioritization of indels when included in a second-pass analysis pipeline.

## Discussion

In most clinical situations when exome sequencing is ordered as a diagnostic test, it is necessary and sufficient to limit the search parameters to well-defined areas of known disease genes and predictable protein changes. However, if such an attempt fails to reveal a molecular diagnosis and an agnostic research effort is made to discover potential new disease genes, non-Mendelian inheritance patterns, multi-gene conditions or unusual pathogenicity, it is desirable to broaden the analytical range to more obscure territories.

Widening search parameters to improve completeness of analysis by including non-exonic variants and medium sized indels may include the “true” variant that may be missed otherwise, but at the expense of increased noise. To handle the additional load of variants, we used the genotype data of 106 unaffected parents as a control cohort, under the assumption that the probands do not share the same rare condition. Although the power of filtration against large control populations, such as 1000 Genomes [30, 31], ESP (NHLBI GO Exome Sequencing Project, URL: http://evs.gs.washington.edu/EVS/) or the ExAC database (Exome Aggregation Consortium, URL: http://exac.broadinstitute.org), is well recognized, we were impressed by the power of filtration that a relatively small cohort of 106 individuals sequenced under the same technical conditions, could provide for analyzing non-exonic variants and medium-sized indels. The use of genome sequencing information released with phase 3 of the 1000 Genomes project will further improve our ability to interpret the biological significance of SNVs and CNVs in non-exonic regions, such as in introns, promoter regions, UTRs, enhancers, intergenic regions and in transcribed non-coding DNA. This is especially important when the search parameters exceed coding regions and canonical splice sites, since most tools to predict deleteriousness are typically limited to these territories.

We characterized the lack of coverage in our exome sequencing studies, which is a generally recognized contributor of false negative results. The application of genome sequencing is known to provide a more homogenous coverage and therefore represents a true advance in the attempt to analyze all genes [32, 33]. However, even this technology lacks coverage at difficult to sequence, possibly non-mappable genomic regions [34]. Other technical advances, such as low amplification technologies (so called “PCR-free chemistry”), which minimizes allele dropout, is only available with larger DNA sample acquisition and whole genome sequencing chemistry.

In current exome sequencing, even highly medically important sets of genes, such as the 56 genes recommended by the American College of Medical Genetics and Genomics for clinical testing, can lack full coverage [35, 36]. Spiking in extra baits enhances the capture of known disease genes. Although coverage has improved with newer capture kits [32], this approach appears inefficient when trying to fully capture all known genes [37], including those not yet associated with disease.

In addition to improved coverage, genome sequencing has also proven useful in the detection of small and large structural variants [38, 39], which may be missed by the standard variant calling pipelines used in exome sequencing. Advances in long read sequencing are hoped to provide optimized identification of structural variants and also allow detection of disease-causing tandem repeats [40–42].

In the future, it will be desirable to include other approaches to variant discovery into an analysis pipeline, beyond classical models of SNVs and CNVs that alter protein function. Examples include changes in DNA methylation [43, 44], disease-causing mobile elements [45] and the spatial organization of the genome [46–48], for example the disruption of long-range enhancer function or perturbation of insulators of topologically associating domains.

Once a research based exome sequencing experiment returns promising variants or novel disease gene candidates, demonstration of causality remains a challenge [10], especially given the typical *n = 1* family situation. Apart from functional studies to elucidate molecular mechanisms, identification of other similarly affected families will be key to validate research findings. Given the limited number of patients seen at a single clinical site, sharing data of both exome sequencing as well as clinical features, especially in standardized form using HPO terms that enable computerized phenotype matching, will become increasingly important [49]. Additionally, comparison of standardized phenotype information to model organisms and integration of pathway analyses have the potential to identify new disease genes for research studies [50–52].

## Conclusions

We explored strategies to improve completeness of analysis in a research setting and demonstrated that the number of variants added is tractable when the search parameters are expanded to include non-coding variants and medium-size indels. Future applications of these approaches will establish to what degree these additional efforts contribute to the number of solved cases, in light of feasibility and efficiency, relative to the number of cases with inconclusive clinical exomes referred for research studies.

## Methods

### Patients

Patients of the UDP were enrolled in clinical protocol 76-HG-0238, “Diagnosis and Treatment of Patients with Inborn Errors of Metabolism and Other Genetic Disorders”, approved by the Institutional Review Board of the National Human Genome Research Institute (NHGRI), and gave written informed consent. Human Phenotype Ontology terms (HPO) were used for standardization as part of clinical phenotyping [12, 53]. The medical records of pediatric UDP applicants reviewed between 2013 and 2015 were screened to determine if exome or genome sequencing had been performed prior to evaluation by the UDP. To estimate family structures, a manually curated pedigree file containing all UDP participants who underwent molecular testing by SNP chip and/or exome sequencing was analyzed.

### Exome sequencing and analysis

DNA was extracted from whole blood using the FlexStar system (Autogen). Libraries of ~280 bp and paired-end index adapters were prepared according to Illumina’s TruSeq V1 or V2 method and sequenced at the NIH Intramural Sequencing center (NISC) on a HiSeq2000 sequencer (Illumina) using 101-bp paired-end reads. Alternatively, 275-325 bp DNA libraries were constructed using KAPA library preparation kit (KAPA), captured with SeqCap EZ Exome plus UTR Lib capture kit (Nimblegen) and sequenced at NISC on a HiSeq2500 (Illumina) using 126-bp paired-end reads. Other exomes were captured using TruSeq kit (Illumina) and sequenced at Axeq (Rockville, MD). For all exome sequencing experiments, short reads were aligned to human reference genome GRCh37 using an in-house developed pipeline based on Novoalign (Novocraft Technologies). Variants were called with HaplotypeCaller and GenotypeGVCFs [54–56]. Annotations utilized snpEff [57] and a combination of internal cohort statistics and publically available data sources (NHLBI GO Exome Sequencing Project (ESP), URL: http://evs.gs.washington.edu/EVS/, 1000Genomes [30]). Basic, computerized variant filtration was used to analyze the effects of family members on the number of variants returned. In brief, rare, non-synonymous, start-gain/loss, stop-gain/loss, frameshift, canonical splice site variants and intronic variants (up to 20 bases from splice sites) were evaluated under homozygous recessive, compound heterozygous, X-linked and *de novo* dominant disease models in families of European descent. A cohort of 54 cases, comprising of the proband and both parents, that were sequenced under the same conditions at NISC using TruSeqV2 capture was used for analysis of low coverage regions, non-coding variants, and medium-sized indels.

### Coverage analysis

Positions within target regions, exons as annotated by the Consensus Coding Sequence project (CCDS) [58–60] or variant positions annotated as disease-causing in the Human Gene Mutation Database (release 2014-1, classes “DM” and “DM?”) [16] were queried for read depth using SAMtools in 54 probands of the UDP [61, 62]. For per base coverage analysis, read depth at each unique position was considered. For exon- and gene-based analysis, the minimum read depth that occurred in a given exon was determined and used to group into categories.

### Non-exonic variants

Non-coding variants outside the regions defined by UCSC exons (annotated in hg19 assembly by the University of California Santa Cruz [63]) were analyzed, while left-normalizing multiallelic variants. The parents of the 54 probands served as a control population, excluding parents of the proband who was examined (*n* = 106 individuals, 212 alleles for autosomal variants, 159 alleles for X-chromosomal variants, and 54 alleles for Y-chromosomal variants). Absence of an alternative allele in the founders was treated as presence of a reference allele. To hypothesize that a variant was too frequently present in the control population to be causative for a rare disease, a cutoff of 6 or more variant alleles was used for autosomal alleles, which corresponds to a minor allele frequency (MAF) of 0.0283 (95 % confidence interval (CI) of 0.013 to 0.0604 using population proportion interval estimation [64]). For X-chromosomal variants, a cutoff at 5 or more alleles was used (MAF of 0.0316, 95 % CI of 0.0136 to 0.0719) and 2 or more variant alleles for Y-chromosomal variants (MAF of 0.037, 95 % CI of 0.0102 to 0.1253). Variants in the probands were annotated with raw and Phred-scaled CADD v1.3 scores [23]. A negative raw score was used to assume that a variant was benign. Phred-scaled CADD scores were used to predict deleteriousness.

### Medium-sized structural variants in exome sequencing

Pindel [29] was used to detect medium-sized structural variants compared to GRCh37 assembly in 54 probands and their parents. The presence of supporting reads was interpreted as a heterozygous variant allele. Structural variants were compared to those called during pipeline genotyping, applying left-normalization of multiallelic variants. Parental data were used to determine the phase of variants in the probands. The parents of 54 probands served as a control population, excluding the parents of the proband who was examined. Under the assumption that all indels identified by Pindel are in heterozygous state, we applied the thresholds as stated above.

## Additional file

Additional file 1: Table S1. Coverage of exons in 54 probands. **Table S2.** Genes with low coverage exons in all 54 probands. (XLSX 9613 kb)

## Abbreviations

UDP: Undiagnosed diseases program
NIH: National Institutes of Health
NHGRI: National Human Genome Research Institute
HPO: Human phenotype ontology terms
NISC: NIH intramural sequencing center
CCDS: Consensus coding sequence project
UCSC: University of California Santa Cruz
MAF: Minor allele frequency
CADD: Combined annotation dependent depletion
HGMD: Human gene mutation database
SNV: Single nucleotide variant
CNV: Copy number variant
SNP: Single nucleotide polymorphism
